# Squamous Cell Carcinoma of the Larynx Arising in Multifocal Pharyngolaryngeal Oncocytic Papillary Cystadenoma: A Case Report and Review of the Literature

**DOI:** 10.1097/MD.0000000000000070

**Published:** 2014-09-05

**Authors:** Markus Stenner, Klaus-Michael Müller, Mario Koopmann, Claudia Rudack

**Affiliations:** Department of Otorhinolaryngology, Head and Neck Surgery (MS, MK, CR); and Institute of Pathology, University Hospital of Münster, Münster, Germany (K-MM).

## Abstract

We report on a rare case of a laryngeal carcinoma arising in a multifocal pharyngolaryngeal oncocytic papillary cystadenoma (OPC). The disease of a 63-year-old man is well documented by computed and positron emission tomography, histology, and electron microscopy. We could show that an OPC can even develop in the pharynx. The coexistence of both tumors makes this a challenging diagnosis for pathologists. Treated by surgery and radiotherapy, both lesions dissolved. Based on the literature available, we discuss the theory that the laryngeal carcinoma might be the result of a true metaplasia facilitated by chronic irritation and recommend a regular follow-up for OPC too. As in benign oncocytic lesions, we could show that the detection of numerous mitochondria is a diagnostic indicator for malignant variants as well.

## INTRODUCTION

Warthin tumor, also known as papillary cystadenoma lymphomatosum, is the second most frequent benign tumor of the salivary glands after pleomorphic adenoma. It is a unique neoplasm composed of oncocytic epithelium with a prominent lymphoid infiltrate. In cases without lymphatic tissue in the subepithelial layer, these tumors are called oncocytic papillary cystadenoma (OPC). OPCs rarely occur in major salivary glands and generally account for <1% of all salivary tumors.^1^ The cytologic features may vary and diagnosis is difficult especially in fine-needle aspiration. Histology mainly shows cystic, oncocytic neoplasms with variable papillary projections. Warthin tumor, oncocytoma, intraductal papilloma, and acinic cell carcinoma may arise in the differential diagnosis. An antimitochondrial monoclonal antibody that recognizes a nonglycosylated mitochondrial protein of 60 kDa in the diagnosis and categorization of salivary tumors recognizes all salivary tumors with oncocytic differentiation.^2^ In pancreatic cancer, oncocytic types of intraductal papillary neoplasms are discussed as precursors for pancreatic cancer.^3^

## CASE REPORT

A 63-year-old man presented to our outpatient department with the diagnosis of a nasopharyngeal carcinoma with ipsilateral neck metastasis. His chief complaint was a progressive indolent swelling of the left neck for 3 weeks. Next, he reported a prolonged dysphonia, having a history of smoking of 30 pack-years. The otorhinolaryngological examination showed a smooth swelling of the left nasopharyngeal recess, a supraglottic mass, a 1-sided vocal cord palsy, and a hard swelling of the left neck. The remaining otorhinolaryngological examination was without any pathologic findings.

**FIGURE 1:**
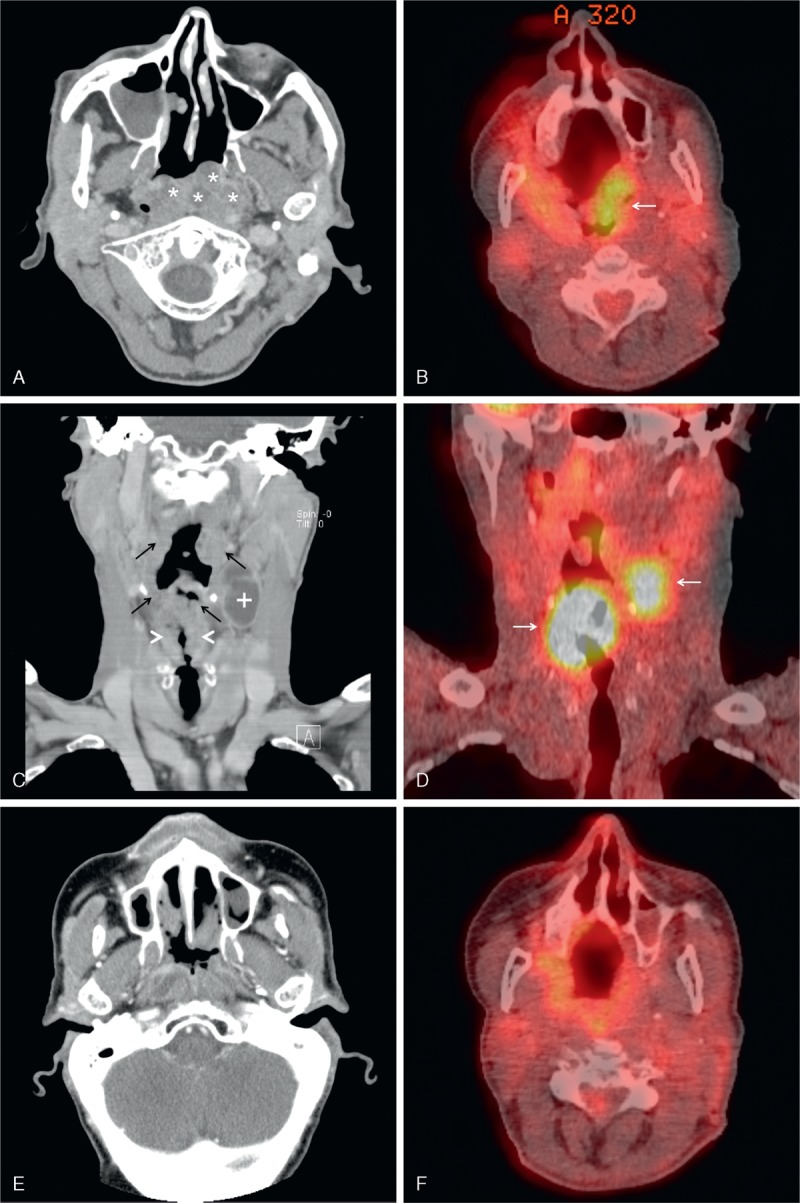
CT (A, C, E) and PET-CT (B, D, F) scans of the patient before (A–D) and after (E and F) therapy. (A) OPC with characteristic cystic lesions (asterisks) of the left epipharyngeal wall. (B) Mild FDG uptake of the OPC (white arrow). (C) OPC of the pharynx (black arrows), swelling of the supraglottic region (white arrowheads), and left-sided neck mass (plus sign). (D) High FDG uptake of supraglottic mass and neck metastasis (white arrows). (E and F) Epipharyngeal wall after therapy. CT = computed tomography, FDG = fluorodeoxyglucose, OPC = oncocytic papillary cystadenoma, PET-CT = positron emission tomography-CT.

**FIGURE 2:**
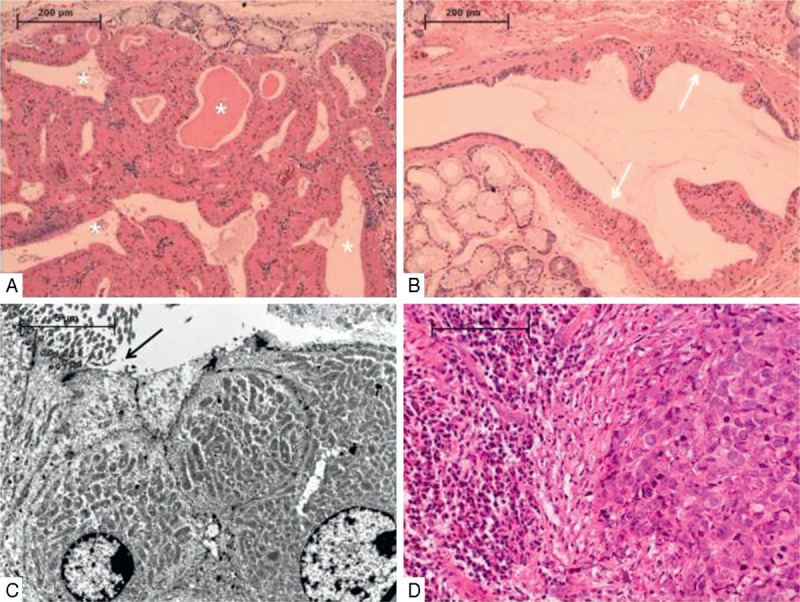
Histopathologic and electron microscopic sections of the patient’ PET-CT lesions. (A) Histologic section of pharyngeal OPC with characteristic focal ductal oncocytic metaplasia and adjacent areas of seromucous glands. Note the microcystic areas (asterisks). (B) Histologic section of pharyngeal OPC with extensive ductal oncocytic metaplasia within a cystically dilated excretory duct (white arrows) and adjacent areas of seromucous glands. (C) Electron microscopy of 3 luminal cells showing a massive mitochondriosis with large numbers of very large, swollen, and closely packed mitochondria. Note the shift from kinocilia cells to oncocytic cells (black arrow). (D) Lymphocytic stroma (left) in direct neighborhood to carcinoma cells (right) of the larynx. OPC = oncocytic papillary cystadenoma, PET-CT = positron emission tomography-CT.

A computed tomography (CT) scan of the head and neck showed an inhomogeneous soft tissue mass of the whole pharyngeal wall with an implied parietal contrast enhancement and cystic lesions (Figure 1A and C). In level II of the left neck, a tumor measuring 3.9 × 2.3 × 2.2 cm with compression of the internal jugular vein could be seen (Figure 1C). Besides that, the laryngeal mucosa appeared irregular with inhomogeneous contrast enhancement (Figure 1C). An endoscopy under general anesthetic was performed and revealed a laryngeal squamous cell carcinoma of both arytenoids and vestibular folds, the laryngeal epiglottis, and the anterior commissure. Beyond that, multiple excisional biopsies (>10) of the whole left pharyngeal wall, the base of the tongue, the tonsils, the soft palate, and the nasopharynx up to 1.3 cm huge and up to 1 cm deep, were without any signs of malignancy. Here, the histopathologic examination showed oncocytic metaplasia in excretory ducts of the small salivary glands with cystadenolymphoma-like lesions in the mucosa of all biopsies. The diagnosis was an OPC (Figure 2A–C). For further exclusion of malignancy, a positron emission tomography-CT (PET-CT) was performed. It showed a left-sided supraglottic fluorodeoxyglucose (FDG) uptake with standardized uptake values (SUVs) of up to 16.3 (reference value of the liver parenchyma 2.1) (Figure 1D). The left-sided neck mass showed an FDG uptake of 8.9 (Figure 1D). The whole left pharyngeal wall showed SUV of up to 5.7 (Figure 1B). The pharyngeal lesion thus was interpreted to be nonmalignant. The general clinical examination as well as vital signs, laboratory evaluation, and an electrocardiogram were without pathologic findings. The patient was free of distant metastasis as evaluated by means of a CT scan of the thorax, an ultrasound examination of the abdomen, and a PET-CT scan. The final clinical staging of the supraglottic laryngeal carcinoma was cT3 cN+ cM0.

A total laryngectomy with a bilateral selective neck dissection was performed. The final diagnosis was a pT3 pN2a (1/59) G3 R0 L1 Pn1 V1 M0 supraglottic laryngeal carcinoma (stage IVA, American Joint Committee on Cancer) (Figure 2D). The margins of excision were clear (7 mm), and the lymph node was without extracapsular spread. The patient received an adjuvant radiation (intensity-modulated radiation therapy) of the tumor region (59.4 Gy) and the lymph drainage ways (50.5 Gy right and 55.8 Gy left). A new CT scan as well as a PET-CT scan 8 months after surgery showed no signs of the OPC (Figure 1E and F), and the neck was without any pathologic findings as well. At the last follow-up 15 months after surgery, the patient was in good health and without any signs of a recurrence.

Institutional approval was given by the head of the department, and informed consent was given by the patient.

## DISCUSSION

To the best of our knowledge, we for the first time present a rare case of a laryngeal carcinoma arising in a multifocal pharyngolaryngeal OPC.

In the larynx, tumors of the benign salivary gland type are rare and less frequent than malignant varieties. Among them, OPC of the larynx has been described since the middle of the last century^4^ and is also referred to as oncocytic cyst, oncocytic papillary cystadenomatosis, oncocytic adenomatous hyperplasia, oxyphil adenoma, oncocytoma, and adenoma in laryngocele. They usually consist of unilocular or multilocular cysts lined by cytologically bland oncocytic epithelium with or without intraluminal papillary ingrowths. Although the nomenclature of these lesions may differ depending on their histologic appearance, these tumors are all generally part of a spectrum of clinically benign cystic and papillary lesions believed to be derived from oncocytic metaplasia and hyperplasia of minor salivary ducts. Most patients are older than 50 years, and complain about hoarseness. The most frequent locations are the false vocal cords and the laryngeal ventricular areas.^5^ Complete endoscopic removal of an OPC is the treatment of choice, because incomplete excision may be associated with recurrence, and thus follow-up is recommended.^6^ In our case, we could show that the OPC dissolved following partial excision by biopsy and radiotherapy. Although the controversy regarding the neoplastic versus degenerative etiology of these lesions could not be resolved from a histopathologic standpoint, their clinical behavior is that of a benign neoplasm. Until now, there exist no reports on OPC of the pharynx. We could show, that an OPC can also become manifest in the form of an extensive multifocal lesion of the larynx and pharynx.

Associated squamous dysplasia in oncocytic cysts of the vocal cords has first been described by Bell et al^7^ in 3 patients. Pathologically this supports the theory of tissue response to chronic irritation, because all 3 patients were heavy smokers. In another case, a patient had multiple oncocytic cysts of the larynx and, concurrently, epidermoid carcinoma of the larynx. The authors histologically documented the multifocal and oncocytic nature of the lesions and interpreted their findings to represent both hyperplastic and metaplastic processes.^8^ Yamase and Putman^9^ reported on a patient with numerous oncocytic lesions of the larynx, in whom laryngectomy for a concurrent epidermoid carcinoma made it possible to study the entire laryngeal mucosa. Here, a hyperplastic component to the oncocytic metaplasia of the ductal epithelium of the seromucinous glands resulted in a spectrum of lesions varying from simple oncocytic cysts to solid lesions that may be erroneously interpreted as true neoplasms. The authors conclude that OPC is a condition affecting the larynx in a diffuse fashion, suggesting the need for follow-up on patients with biopsy-proven oncocytic lesions.^9^ On the contrary, a case of multifocal laryngeal cystic oncocytic hyperplasia necessitating laryngectomy is reported. A CT scan of the larynx suggested the destruction of the cartilage. Nevertheless, malignant histologic features were not present.^10^ In our case, even though we could document by histology the direct vicinity of oncocytic lesions to carcinoma cells (Figure 2D), we cannot logically support any argument that these tumors have any causal relationship with each other. Because we did not see concrete transitions from oncocytic to carcinoma cells we support the theory of a collision tumor. Thus, the 2 tumors may influence each other in terms of a chronic irritation. Whether a toxic irritation facilitates the metaplasia or not may be speculated. Nevertheless, this is an important reason for a regular follow-up of such patients. Another possible explanation for the histologic changes seen might be an obstructive change in minor salivary gland lobules as a result of a mass effect from the primary carcinoma, that is, metaplasia, a common reactive finding following obstruction.

In oncocytic carcinoma as well as in malignant papillary cystadenoma lymphomatosum, antimitochondrial antibody positivity is known to be evident in the cytoplasm of the tumor cells. Electron microscopically, large numbers of very large, swollen, and closely packed mitochondria can be observed in almost all the tumor cells. In our case, we could show the same for a carcinoma arising in an OPC (Figure 2C). Thus, immunohistochemistry using an antimitochondrial antibody and electron microscopy is useful and helpful for the diagnosis of oncocytic lesions as OPC.

## CONCLUSION

This rare case of a laryngeal carcinoma arising in a multifocal pharyngolaryngeal OPC shows that an OPC can develop in the pharynx as well. We discussed the theory that the carcinoma is the result of a true metaplasia that may be facilitated by toxic irritation. Although the OPC was treated by excisional biopsy and radiotherapy, a regular follow-up should be recommended for all patients presenting with an OPC. As in benign oncocytic lesions, the detection of large numbers of mitochondria is a diagnostic indicator for malignant variants as well.
